# Characterization technique of gases permeation properties in polymers: H_2_, He, N_2_ and Ar gas

**DOI:** 10.1038/s41598-022-07321-1

**Published:** 2022-02-28

**Authors:** Jae Kap Jung, Ji Hun Lee, Jin Sub Jang, Nak Kwan Chung, Chang Young Park, Un Bong Baek, Seung Hoon Nahm

**Affiliations:** 1 Team of Hydrogen Energy Materials Research, Korea Research Institute of Standards and Science, Daejeon, 34113 Korea; 2 Department of Physics and Research Institute of Natural Science, Gyeongsang National University, Jinju, 52828 Korea; 3 Team of Hydrogen Energy Materials Research, Korea Research Institute of Standards and Science, Daejeon, 34113 Korea

**Keywords:** Energy science and technology, Physics

## Abstract

We demonstrate a simple experimental technology for characterizing the gas permeation properties of H_2_, He, N_2_ and Ar absorbed in polymers. This is based on the volumetric measurement of released gas and an upgraded diffusion analysis program after high-pressure exposure. Three channel measurements of sorption content of gases emitted from polymers after decompression are simultaneously conducted, and then, the gas uptake/diffusivity as a function of exposed pressure are determined in nitrile butadiene rubber (NBR), ethylene propylene diene monomer (EPDM) rubbers, low-density polyethylene (LDPE) and high-density polyethylene (HDPE), which are used for gas sealing materials under high pressure. The pressure-dependent gas transport behaviors of the four gases are presented and compared. Gas sorption follows Henry’s law up to 9 MPa, while pressure-dependent diffusion behavior is not observed below 6 MPa. The magnitude of the diffusivity of the four gases decreases in the order *D*_He_ > *D*_H2_ > *D*_Ar_ > *D*_N2_ in all polymers, closely related to the kinetic diameter of the gas molecules. The dependence of gas species on solubility is in contrast to that on diffusivity. The linear correlation between logarithmic solubility and critical temperature of the gas molecule was newly observed.

## Introduction

Permeation is the penetration process of a permeant such as a liquid, gas, or vapor through the material membrane of a solid. Permeation comprises three processes: adsorption of the permeating species into the polymer, diffusion through the polymer membrane and desorption of the permeating species from the polymer surface. Permeation is important for many design applications, such as packaging, gas separation, analytical chemistry, polymer electrolytes, and biosensors^1–6^. Studying the permeability of gases through materials under different environmental conditions is crucial to understand if the corresponding material is adapted to the chosen gases. Specifically, gas selectivity and permeation characteristics such as solubility, diffusivity and permeability are important requirements for appropriate polymer membrane selection. There are diverse methods in which the permeation of a material can be measured. These methods include manometric methods^7,8^, constant-pressure methods^7^, gravimetric techniques^9^, magnetic suspension balance methods^10,11^, gas chromatography (GC)^12^ and numerical simulation^13,14^.

The differential pressure method called as manometric method is tested following the ASTM D143 standard. The permeation parameters are determined by measuring both the permeation and diffusivity of a specimen plate placed between chamber with a feed and permeation sides via monitoring pressure versus elapsed time. However, important factors such as high vacuum circumstance before stating the measurement, a limited sample shape/dimension, leakage in measuring cell and outgassing from volatile specimen should be considered.

GC is an advanced technique requiring the complicated process and pre-calibration for determining hydrogen permeability from individual GC peaks in electrical units of pA s. This method can precisely quantify the amount of hydrogen uptake in even small amounts of specimen because of good resolution of 0.01 wt ppm. However, the technique seems not be effective method.

Gravimetric technique by electronic balances is very sensitive to environment of temperature and humidity because it detects small changes in electrical resistance, which is proportional to the acting deforming force following the principle of a Wheatstone bridge circuit. Thus, this measurement is a very sensitive technique depending on the effect of the offset of electronic balances and on the stability of the temperature/humidity in the laboratory. This method maintains traceability because the electronic balance can be calibrated by using standard weights traceable to national standards. Magnetic suspension balance method is utilized for in-situ measurement of specimen under high pressure. In summary, most methods are time-consuming processes with complicated processes and fine control.

Therefore, effective and easy measurement is required to enhance the reliability of permeability characteristics. An effective technique in present work is to combine a volumetric measurement using a graduated cylinder and upgraded diffusion analysis program. We have confirmed the volumetric analysis technique (VAT) in previous researches^15^ comparing the results obtained by VAT with those by different methods, such as gas chromatography by thermal desorption analysis, gravimetric measurement by electronic balance for same samples. The results are found to be consistent with each other. In addition, the developed technique reduces the uncertainty of permeation parameters by varying the temperature and pressure of the laboratory environment by compensating the variations. The advantage of the technique is also commonly applied for determination of various gas permeation parameters with simultaneous parallel measurements more than three specimens, regardless of gas species and shape/dimension of the specimen. The techniques were applied to nitrile butadiene rubber (NBR) and ethylene propylene diene monomer (EPDM) polymers, which are used for gas sealing materials. The use of specimens of high-density polyethylene (HDPE) as liner materials of a type IV tank in a fuel cell electric vehicle and low-density polyethylene (LDPE) as plastics was also included for experimental investigation.

The aim of this paper was to present precise data on the gas permeability characteristics of polymer materials. The solubility, diffusivity and permeability of the four polymers were investigated as a function of the exposed pressure and gas species such as H_2_, He, N_2_ and Ar. The solubility and diffusivity in NBR, EPDM, LDPE and HDPE polymers could be correlated in terms of the kinetic diameter and critical temperature of the molecule in the gases employed. The uncertainty analysis against the measured data is carried out in order that the method could be applicable as a standard test for the permeation properties for various gases of polymers which is used as a gas sealing materials under the high pressure.

## Experimental aspects

### Sample preparation and gas exposure condition

The compositions and densities of the NBR and EPDM polymer specimens used in this study are already listed in previous literature^15,16^. Heat treatment of the polymer is performed at 60 °C for 48 h to minimize outgassing from the rubber. For the volume dependence on the permeation parameter, NBR and EPDM specimens are used as following shapes/dimensions:

- cylindrical NBR with a radius of 7.0 mm and thicknesses of 1.1 mm and 2.2 mm
- spherical NBR with a radius of 5.0 mm
- cylindrical EPDM with a radius of 7.0 mm and thicknesses of 1.4 mm and 2.5 mm
- spherical EPDM with a radius of 4.9 mm

Additionally, two types of polyethylene fabricated at King Plastic Corporation with advanced antimicrobial technology were employed in the experimental investigations. The physical and mechanical properties of LDPE and HDPE specimens are presented in Table 1. To study volume dependence on the permeation parameter, LDPE and HDPE specimens with different shapes/dimensions were prepared as follows:

- LDPE rectangular plane sheet with a length of 15.0 mm, a width of 15.0 mm and thicknesses of 2.2 mm and 3.1 mm
- HDPE rectangular plane sheet with a length of 15.0 mm, width of 15.0 mm and thickness of 2.4 mm
- spherical HDPE with a radius of 4.8 mm

**Table 1:** Physical and mechanical properties of LDPE and HDPE specimens.

Material	LDPE	HDPE
Fabrication	Compression molding	Compression molding
Density (g/ml)	0.86	0.91
Tensile strength (psi)	More than 1400	More than 4100
Hardness (Shore D)	42	68
Tensile modulus (psi)		255,000
Flexural modulus (psi)	30,000	185,000
Coefficient of thermal expansion (in./in./°F)	6 × 10^–5^	6 × 10^–5^ ~ 7.9 × 10^–5^

An SUS 316 chamber with an inner diameter of 50 mm and height of 90 mm was used for gas exposure to high pressure at room temperature and the specified pressure. The chamber was purged three times with the corresponding gas at 1 ~ 3 MPa depending on the exposed pressure before gas exposure. We exposed the gas for 24 h to the specimen in the pressure range from 1.5 to 10 MPa. Gas charging for 24 h is sufficient to attain the equilibrium state for gas sorption, except for N_2_ gas exposure. N_2_ gas charging for 48 h is needed to attain the equilibrium state for N_2_ sorption, because of its slow diffusion. After exposure to gas, the valve was opened, and the gas in the chamber was released. After decompression, the elapsed time was recorded from the moment (*t* = 0) at which the high-pressure gas in the chamber was reduced to atmospheric pressure when the time was set to zero. Since the specimen was loaded in the graduated cylinder after decompression, it took approximately 5 ~ 10 min to start the measurement. The gas content emitted for the inevitable time lag could be measured by offset determination.

## Volumetric analysis measurement system

### Volumetric measurement of emitted gas

**Figure 1:**
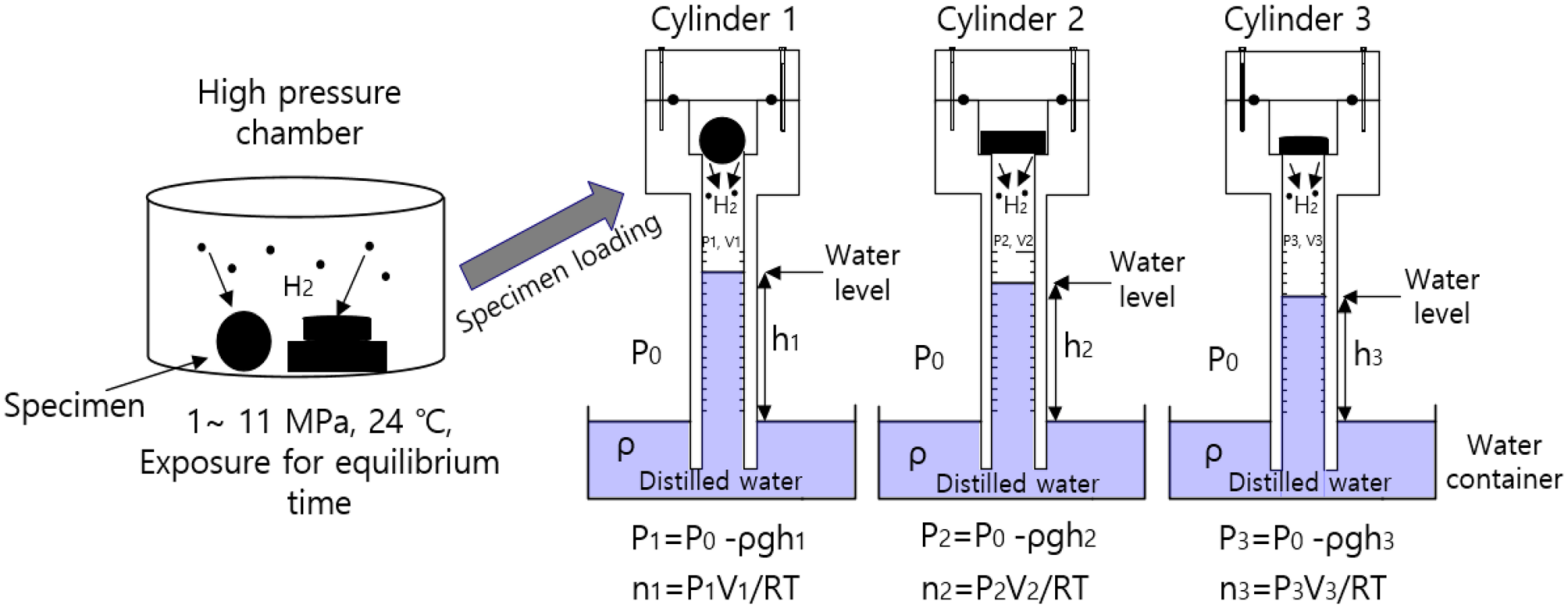
Schematic diagram of the three-channel volumetric measurement system in which three cylinders are standing. The blue part indicates the distilled water filled in water containers and cylinders.

Figure 1a shows the three-channel volumetric measurement system with three graduated cylinders used to measure the released gas. After exposure to the high-pressure chamber and subsequent decompression, the specimen is loaded into the gas space of a graduated cylinder. Three parallel standing graduated cylinders immersed partially in each water container collect and measure the gas released from the specimen. The temperature and pressure measured near the sample are applied for the calculation of gas uptake.

The pressures (${P}_{1}, {P}_{2}, \; {and \; P}_{3}$) inside each graduated cylinder for the three channels are expressed as^16^.

1 $${P}_{1}={P}_{o}-\rho g{h}_{1}, {P}_{2}={P}_{o}-\rho g{h}_{2}, {P}_{3}={P}_{o}-\rho g{h}_{3}.$$

${P}_{o}$ is the outside atmosphere pressure of the cylinder, $\uprho$ is the density of distilled water in the water container, and *g* is gravity. ${h}_{1}$, ${h}_{2}$ and ${h}_{3}$ are the heights of the distilled water level inside the corresponding graduated cylinder measured from the water levels in the water containers of cylinder 1, cylinder 2 and cylinder 3, respectively. ${V}_{1}, {V}_{2} \; \mathrm{and} \; {V}_{3}$ are the gas volumes inside the corresponding graduated cylinder filled with gas. As shown in Fig. 1, the gas inside the cylinder is governed by the ideal gas equation, *PV* = *nRT*, and $R$ is the gas constant of 8.20544 × 10^–5^ m^3^ atm/(mol K).

The total number of moles (${n}_{1}$, ${n}_{2} \; \mathrm{and} \; {n}_{3}$) of gas inside the corresponding cylinder is expressed at specified *P* and *T* as follows for the three cylinders.

2 $${n}_{1}={n}_{\mathrm{1,0}}+\Delta {n}_{1}=\frac{{(P}_{o}-\rho g{h}_{1}){V}_{1}}{RT}, {n}_{2}= {n}_{\mathrm{2,0}}+\Delta {n}_{2 }=\frac{{(P}_{o}-\rho g{h}_{2}){V}_{2}}{RT}, {n}_{3}= {n}_{\mathrm{3,0}}+\Delta {n}_{3}=\frac{{(P}_{o}-\rho g{h}_{3}){V}_{3}}{RT}.$$

${n}_{\mathrm{1,0}}$, ${n}_{\mathrm{2,0}}$ and ${n}_{\mathrm{3,0}}$ are the initial mole numbers of air already in cylinder 1, cylinder 2 and cylinder 3, respectively, before gas emission. The gas released from the specimen after decompression lowers the water level of the cylinder. Thus, the increased number of moles ($\Delta {n}_{1}, \Delta {n}_{2} \; \mathrm{and} \; \Delta {n}_{3}$) of emitted gas in each cylinder after decompression is obtained by measuring the increase in volume (${\Delta V}_{1}, {\Delta V}_{2} \; \mathrm{ and } \; {\Delta V}_{3})$ in each graduated cylinder, i.e., lowering of the water level as follows.

3 $${\Delta n}_{1}=\frac{{(P}_{o}-\rho g{h}_{1}){\Delta V}_{1}}{RT}, {\Delta n}_{2}=\frac{{(P}_{o}-\rho g{h}_{2}){\Delta V}_{2}}{RT}, {\Delta n}_{3}=\frac{{(P}_{o}-\rho g{h}_{3}){\Delta V}_{3}}{RT}.$$

The increased number of moles in each cylinder is converted to the corresponding mass concentration [${C}_{1}\left(\mathrm{t}\right), {C}_{2}\left(\mathrm{t}\right)\mathrm{ and }{C}_{3}(\mathrm{t})]$ of gas emitted from the rubber sample as follows.

4 $$\begin{aligned} &{C}_{1}\left(\mathrm{t}\right)\left[\mathrm{wt}\cdot \mathrm{ppm}\right]=\Delta {n}_{1}\left[\mathrm{mol}\right]\times \frac{{m}_{gas} \left[\frac{\mathrm{g}}{\mathrm{mol}}\right]}{{m}_{sample}\left[\mathrm{g}\right]}\times {10}^{6} \\ & {C}_{2}\left(\mathrm{t}\right)\left[\mathrm{wt}\cdot \mathrm{ppm}\right]=\Delta {n}_{2}\left[\mathrm{mol}\right]\times \frac{{m}_{gas} \left[\frac{\mathrm{g}}{\mathrm{mol}}\right]}{{m}_{sample}\left[\mathrm{g}\right]}\times {10}^{6} \\ & {C}_{3}\left(\mathrm{t}\right)\left[\mathrm{wt}\cdot \mathrm{ppm}\right]=\Delta {n}_{3}\left[\mathrm{mol}\right]\times \frac{{m}_{gas} \left[\frac{\mathrm{g}}{\mathrm{mol}}\right]}{{m}_{sample}\left[\mathrm{g}\right]}\times {10}^{6}. \end{aligned}$$

${m}_{gas}$[*g*/mol] is the molar mass of the gas investigated. For example, for H_2_ gas, ${m}_{H2 gas}$ is 2.016 g/mol. ${m}_{sample}$ is the mass of the specimen. By measuring the change in the water level ($\Delta V$), the increased mole number is obtained, and thus, the mass concentration of the emitted gas is transformed by Eq. (4). Therefore, the time-dependent mass concentration of the released gas is obtained by measuring the water level change, $\Delta V,$ versus the elapsed time after decompression.

### Time-dependent emitted gas concentration versus specimen shape

The adsorption of gas at high-pressure causes the release of gas dissolved in rubber after decompression to atmospheric pressure. Assuming that the adsorption and desorption of gas is a diffusion-controlled process, the emitted gas concentration ${C}_{E}(t)$ in the desorption process is expressed as^17,18^

5 $${C}_{E}\left(t\right)={C}_{\infty }\left[1-\frac{6}{{\pi }^{2}}\sum_{n=1}^{\infty }\frac{1}{{n}^{2}}\mathrm{exp}\left(-\frac{D{n}^{2}{\pi }^{2}t}{{a}^{2}}\right)\right].$$

Equation (5) is the solution to Fick’s second diffusion law for a spherical sample with an initially constant uniform gas concentration and constant concentration at the spherical surface. ${C}_{\infty }$ is the saturated gas mass at an infinitely long time, i.e., the total emitted mass concentration or gas uptake in the adsorption process. *D* is the diffusion coefficient of desorption. $a$ is the radius of the spherical rubber^17,18^.

Under the boundary condition, i.e., the uniform gas concentration is initially maintained, and assuming the cylindrical surfaces are kept at a constant concentration, the emitted gas content ${C}_{E}(t)$ for a cylindrical specimen is expressed as^17,18^

6 $$\frac{{C}_{E}\left(t\right)}{{C}_{\infty }}=1-\frac{32}{{\pi }^{2}}\times \left[\sum_{n=0}^{\infty }\frac{exp\left\{\frac{{-\left(2n+1\right)}^{2}{\pi }^{2}Dt}{{l}^{2}}\right\}}{{\left(2n+1\right)}^{2}}\right]\times \left[\sum_{n=1}^{\infty }\frac{exp\left\{-\frac{D{\beta }_{n}^{2}t}{{\rho }^{2}}\right\}}{{\beta }_{n}^{2}}\right].$$

In Eq. (6), $l$ is the thickness of the cylindrical rubber sample, $\rho \mathrm{is}$ the radius, and ${\beta }_{n}$ is the root of the zero-order Bessel function.

Similarly, Eq. (7) is the solution to Fick’s second diffusion law for a plane sheet specimen with an initially constant uniform hydrogen concentration and constant concentration at the surface.

7 $${C}_{E}\left(t\right)={C}_{\infty }\left[1-\frac{8}{{\pi }^{2}}\sum_{n=0}^{\infty }\frac{1}{{\left(2n+1\right)}^{2}}\mathrm{exp}\left(-\frac{D{\left(2n+1\right)}^{2}{\pi }^{2}t}{{T}^{2}}\right)\right].$$

*T* is the thickness of the sheet-shaped rubber for the emitted gas content ${C}_{E}(t)$

To analyze the mass concentration data, we used a upgraded diffusion analysis program developed using Visual Studio to calculate *D* $\mathrm{and}$ ${C}_{\infty }$ in Eqs. (5), (6) and (7) based on least-squares regression^16,19^. A diffusion analysis program is updated for use of specimen with spherical, cylindrical and sheet shapes at both modes of residual and emission mode.

## Results and discussion

### Measured example of diffusivity and uptake determined for four gases and polymers

**Figure 2:**
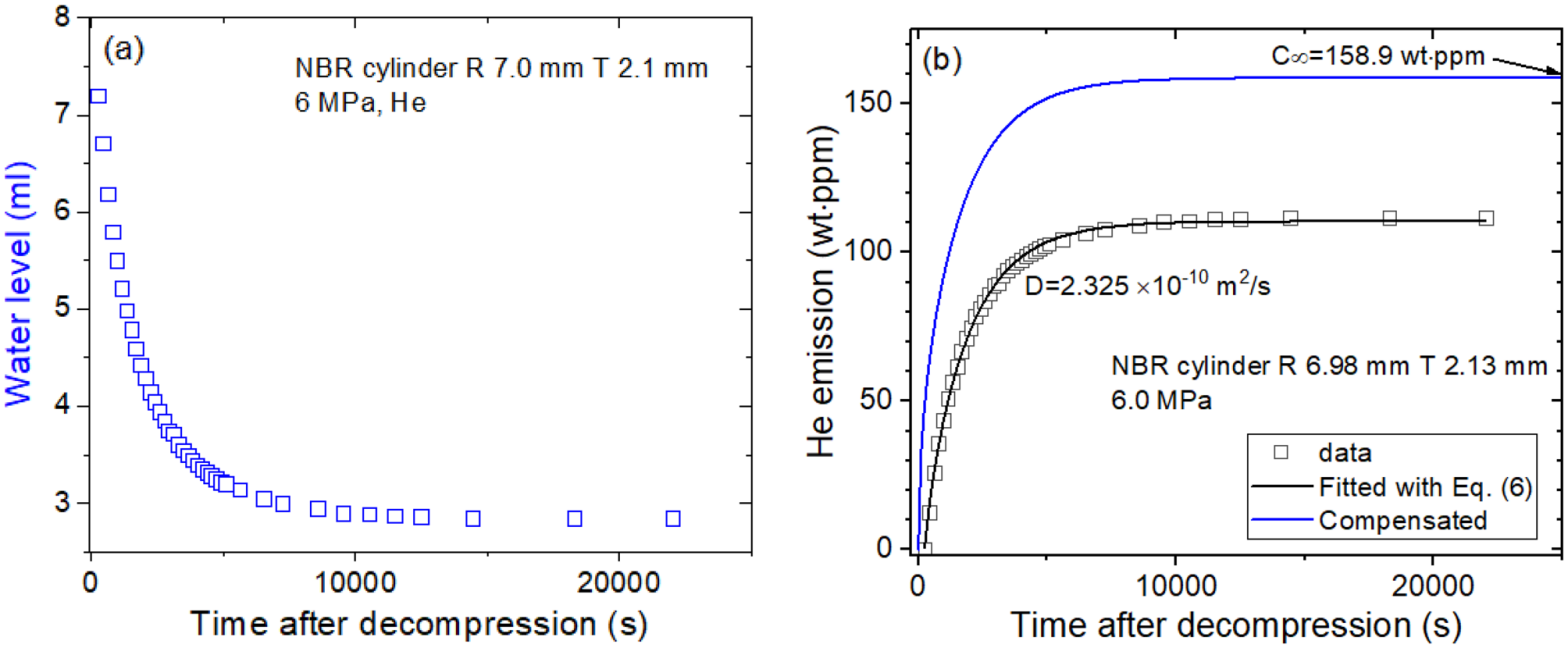
Sequence for obtaining the diffusion parameter in NBR cylindrical rubber. (**a**) Water level versus time after decompression and (**b**) time-varying He mass concentration transformed from the water level. As a result of the experimental investigation, the diffusion parameters D and C_∞_ are determined using a diffusion analysis program. The blue line is the total compensated emission curve restoring the missing content for lag time. R and T indicate the radius and thickness, respectively, of the cylindrical specimen.

The gas emitted from the specimen lowers the water level, which exponentially decreases with increasing elapsed time. By the application of Eqs. (3) to (7), the water level is transferred to mass concentration, and then, the gas uptake and diffusivity are determined via diffusion analysis program. Figures 2, 4 and 5 show the example of sequence used to obtain the diffusion parameters of four different gases for NBR, EPDM, LDPE and HDPE.

Figure 2a shows the time-varying water level measured directly by He gas emitted from the NBR cylindrical specimen. The water level as a function of time is transferred to mass concentration by Eqs. (3) and (4). Figure 2b shows diffusion parameters *D* $\mathrm{and}$ ${C}_{\infty }$, determined using a diffusion analysis program. *D* $\mathrm{and}$ ${C}_{\infty }$ are found to be 2.325 × 10^–10^ m^2^/s and 158.9 wt ppm, respectively, at 6.0 Ma and 297 K. The emitted He content is saturated above 10,000 s, and total He uptake is taken by extrapolation to restore the missing content for lag time. Fast diffusivity is unexpectedly observed for He gas.

**Figure 3:**
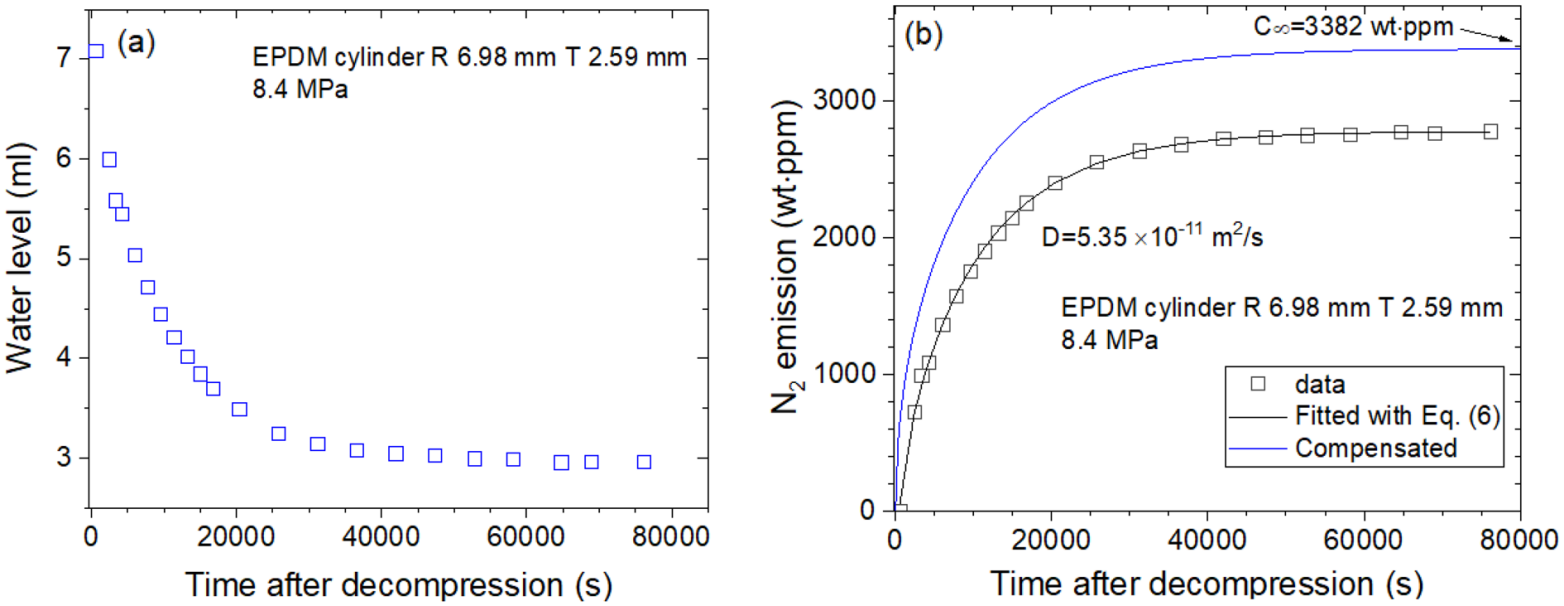
Sequence for obtaining the diffusion parameter in EPDM cylindrical rubber. (**a**) Water level versus time after decompression and (**b**) time-varying N_2_ mass concentration transformed from the water level. As a result of the experimental investigation, the diffusion parameters D and C∞ are determined using a diffusion analysis program. The blue line is the total compensated emission curve restoring the missing content for lag time. R and T indicate the radius and thickness, respectively, of the cylindrical specimen.

Figure 3a shows the time-varying water level measured directly by N_2_ gas emitted from EPDM. The water level as a function of time is transformed to mass concentration. Figure 3b shows diffusion parameters D and C_∞_, obtained from the mass content determined using a diffusion analysis program. D and C_∞_ are found to be 5.35 × 10^–11^ m^2^/s and 3382 wt ppm, respectively, at 8.4 MPa and 297 K. The emitted N_2_ content is saturated above 40,000 s, and total N_2_ uptake is also taken by extrapolation to restore the missing content for lag time. A relatively slow diffusivity is observed for N_2_ compared to He gas.

**Figure 4:**
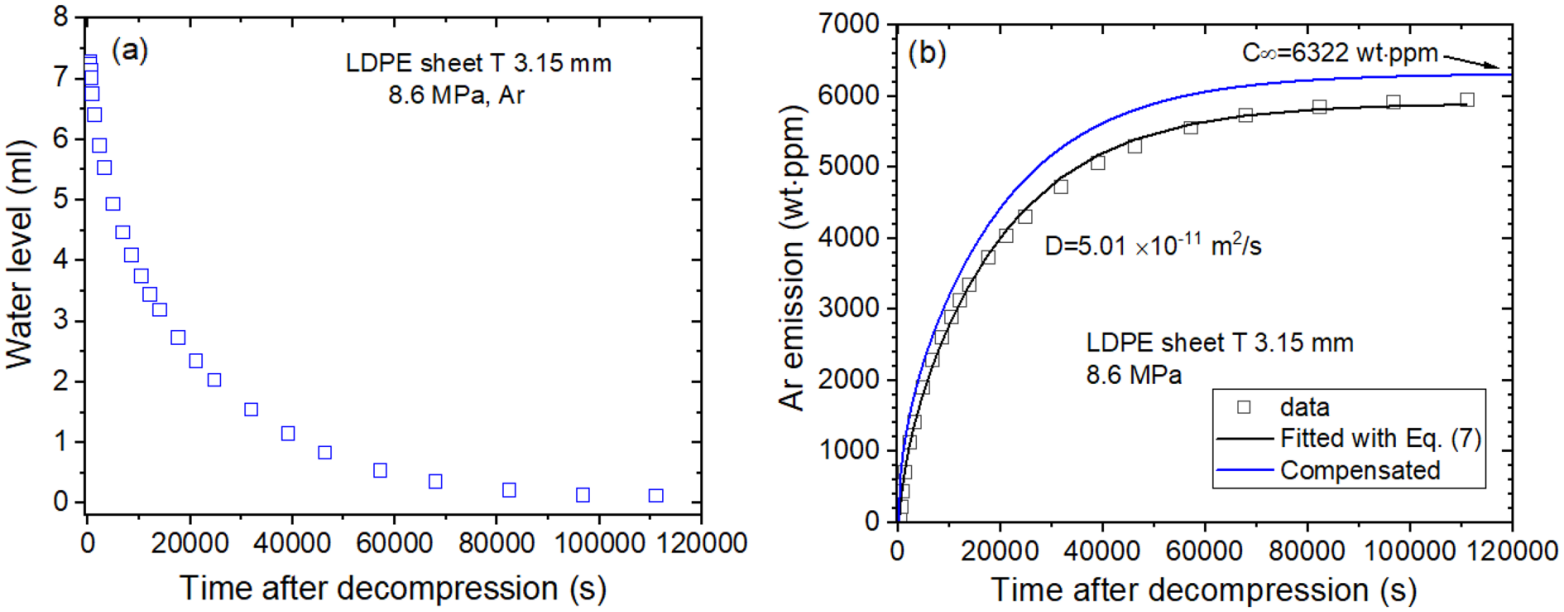
Sequence for obtaining the diffusion parameter in the LDPE sheet. (**a**) Water level versus time after decompression and (**b**) time-varying Ar mass concentration transformed from the water level. As a result of the experimental investigation, the diffusion parameters D and C_∞_ are determined using a diffusion analysis program. The blue line is the total compensated emission curve restoring the missing content for lag time. T indicates the thickness of the sheet specimen.

Figure 4a shows the time-varying water level measured directly by Ar gas emitted from LDPE. The water level as a function of time is transformed to mass concentration. Figure 4b shows diffusion parameters D and C_∞_, which are found to be 5.01 × 10^–11^ m^2^/s and 6322 wt ppm, respectively, at 8.6 MPa and 297 K. The emitted Ar content is saturated above 100,000 s, and total Ar uptake is taken by extrapolation to restore the missing content for lag time.

**Figure 5:**
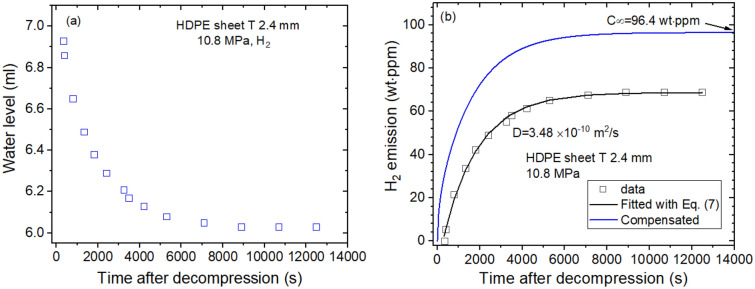
Sequence for obtaining the diffusion parameter in the HDPE sheet. (**a**) Water level versus time after decompression and (**b**) time-varying H_2_ mass concentration transformed from the water level. As a result of the experimental investigation, the diffusion parameters D and C_∞_ are determined using a diffusion analysis program. The blue line is the total compensated emission curve restoring the missing content for lag time. T indicates the thickness of the sheet specimen.

Figure 5a shows the time-varying water level measured directly by H_2_ gas emitted from HDPE. The water level as a function of time is transformed to mass concentration. Figure 5b shows diffusion parameters D and C_∞_, which are found to be 3.48 × 10^–10^ m^2^/s and 96.4 wt ppm, respectively, at 10.8 MPa and 297 K. The emitted H_2_ content is saturated above 10,000 s, and total H_2_ uptake is taken by extrapolation to restore the missing content for lag time. The gas uptake and diffusivity of the four gases have characteristic properties.

### Pressure dependence on the permeation parameter

**Figure 6:**
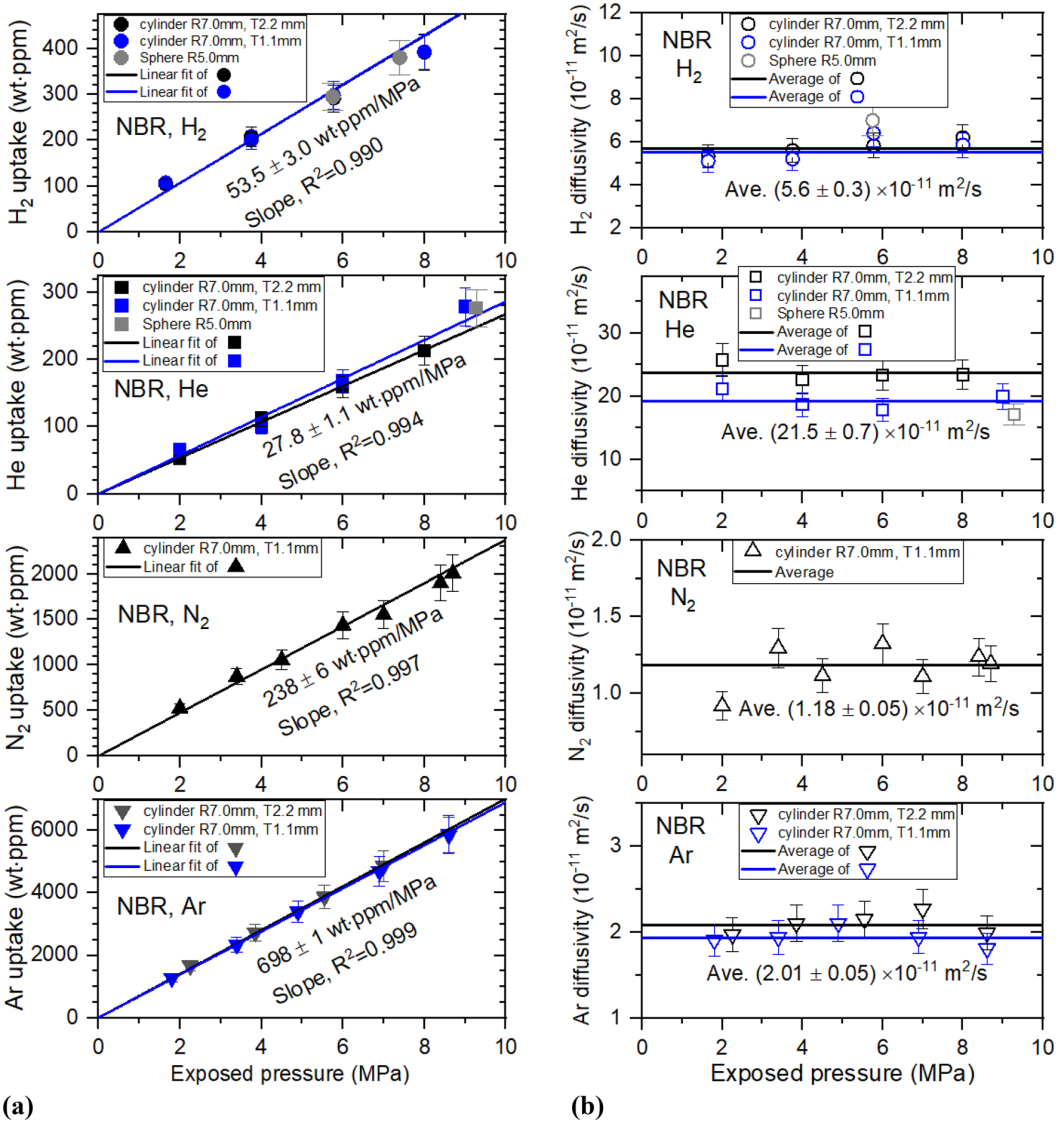
(**a**) Gas uptake (${C}_{\infty }$) and (**b**) diffusivity (*D*) versus exposed pressure for four gases in cylindrical-shaped NBR with different thicknesses. R and T indicate the radius and thickness, respectively, of cylindrical NBR.

**Figure 7:**
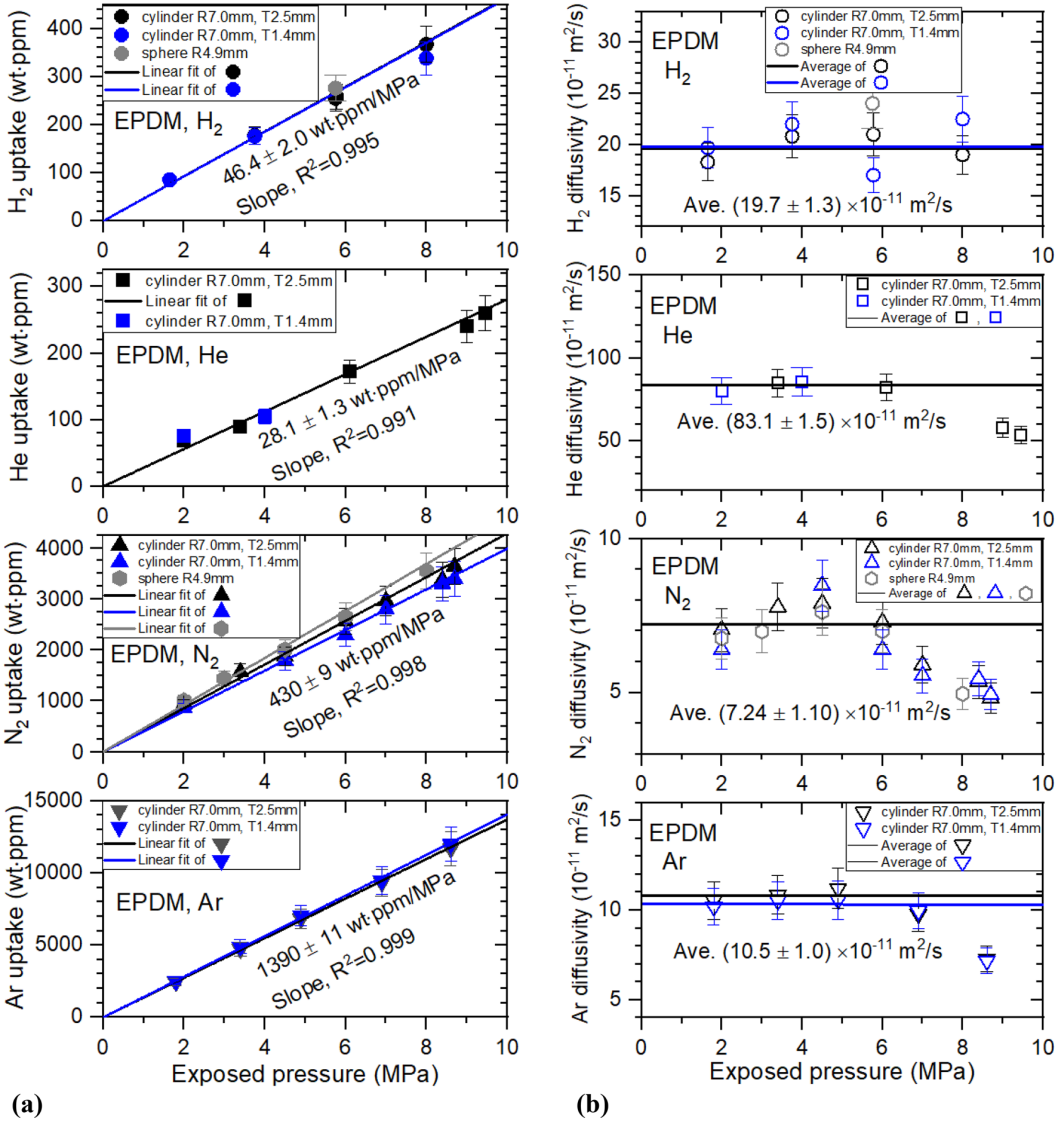
(**a**) Gas uptake (${C}_{\infty }$) and (**b**) diffusivity (*D*) versus exposed pressure for four gases in cylindrical EPDM with different thicknesses and spherical EPDM. R indicates the radius of cylindrical and spherical EPDM. T indicates the thickness of cylindrical EPDM.

**Figure 8:**
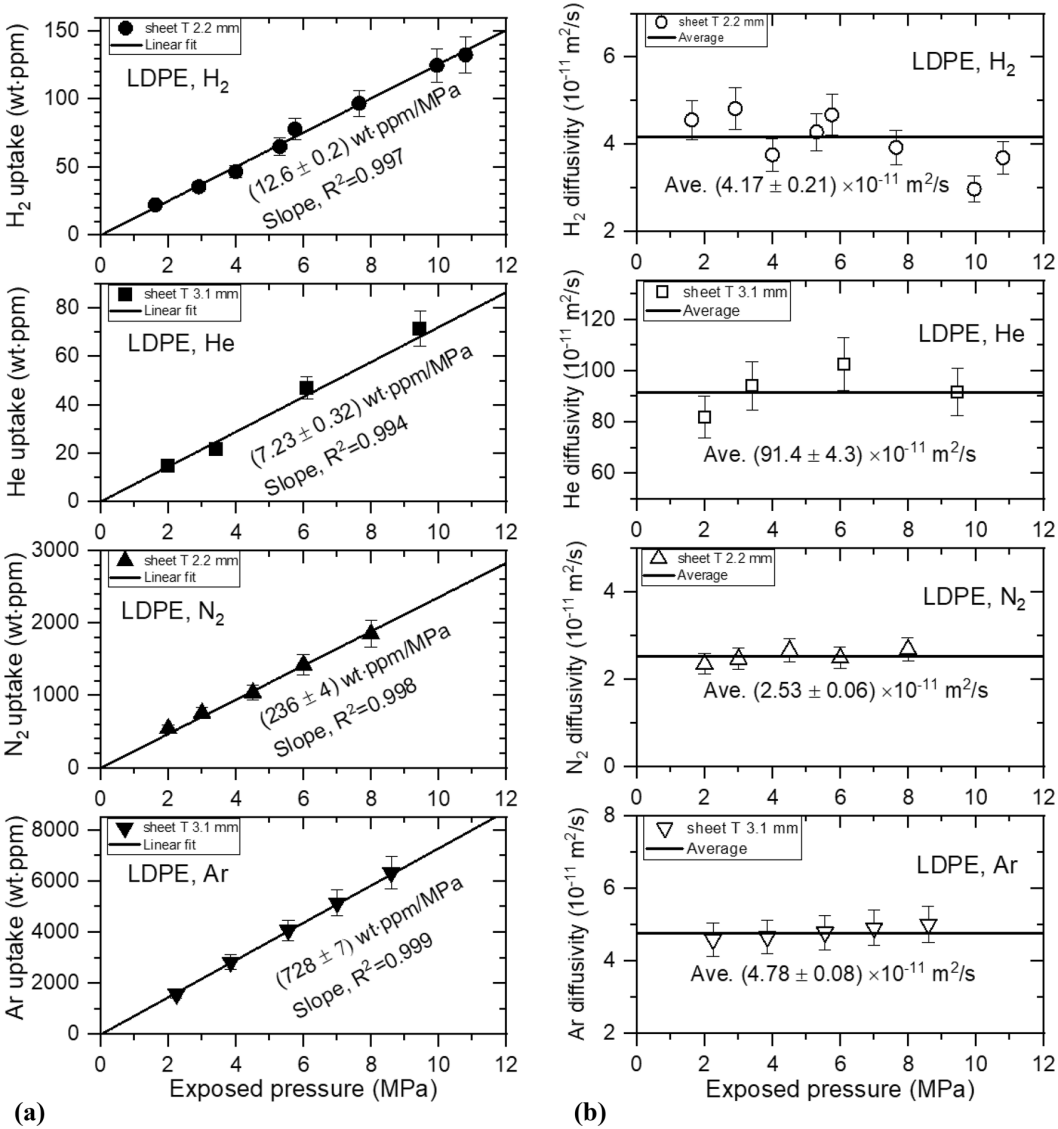
(**a**) Gas uptake (${C}_{\infty }$) and (**b**) diffusivity (*D*) versus exposed pressure for four gases in LDPE sheets with different thicknesses. T indicates the thickness of the LDPE sheet.

**Figure 9:**
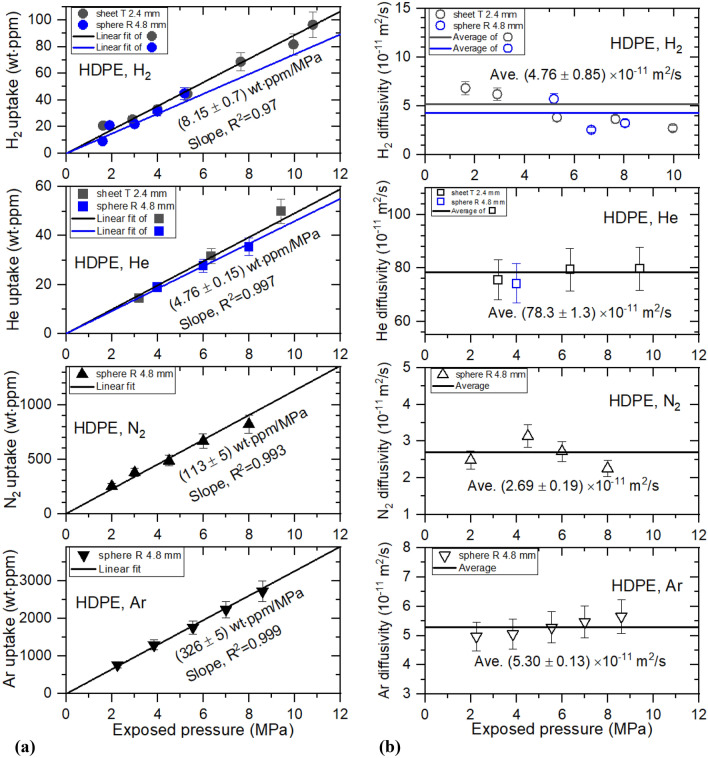
(**a**) Gas uptake (${C}_{\infty }$) and (**b**) diffusivity (*D*) versus exposed pressure for four gases in HDPE sheets with different thicknesses and HDPE spheres. T indicates the thickness of the HDPE sheet. R indicates the radius of the HDPE sphere.

Figures 6, 7, 8 and 9 show permeation parameters versus exposed pressure in NBR, EPDM, LDPE and HDPE for four different gases. The diffusion parameters ${C}_{\infty }$ and D are determined using a diffusion analysis program by the application of Eqs. (5) to (7) based on least-squares. The standard deviation between the experimental data and the diffusion model was within 3% for all polymers.

All gas uptake follows Henry’s law^20^ up to 9 MPa in NBR, with a squared correlation coefficient R^2^ ˃ 0.990, as indicated by the black and blue lines of Fig. 6a, implying that gas does not dissociate and penetrates into the specimen as a gas molecule. The slopes obtained for the specimen indicate Henry’s law solubility. As shown in Fig. 6b, the diffusivity does not present distinct pressure dependence. The error bars indicate a relative expanded uncertainty of 10%, as evaluated in previous research. Thus, we take average diffusivity as diffusivity, indicated as black and blue horizontal lines.

All gas uptake follows Henry’s law up to 9 MPa in EPDM, with R^2^ ˃ 0.991, as indicated by the black, blue and gray lines of Fig. 7a. Additionally, Fig. 7b shows that diffusivity decreases with increasing pressure above 6 MPa, except for H_2_ diffusivity. This may be ascribed to bulk diffusion associated with the mean free path, normally observed for high-pressure gas diffusion. At pressures below 6 MPa in Fig. 7b, we also take the average diffusivity as the representative diffusivity, indicated by black and blue horizontal lines. As shown in Figs. 6 and 7, thickness dependence on permeation parameters in cylindrical-shaped NBR and EPDM was not observed, as expected.

All gas uptake follows Henry’s law up to 9 MPa in LDPE, with R^2^ ˃ 0.994, as indicated by the black lines of Fig. 8a. As shown in Fig. 8b, the diffusivity does not exhibit distinct pressure dependence. Thus, we take the average diffusivity, represented by black horizontal lines.

All gas uptake follows Henry’s law up to 9 MPa in HDPE, with R^2^ ˃ 0.97, as indicated by the black and blue lines of Fig. 9a. The relatively larger correlation coefficient in HDPE than in the other three polymers is attributed to the increased uncertainty due to the small gas solubility and fast diffusion. As shown in Fig. 9b, the diffusivity does not represent exhibit pressure dependence. Thus, we also take the average diffusivity, represented by black and blue horizontal lines. Thickness and shape dependence of the permeation parameters in HDPE was not observed within the expanded uncertainty.

Meanwhile, the solubility (*S*) is determined from the linear slope obtained in Figs. 6a, 7, 8 and 9a as follows.

8 $$S\left[\frac{mol}{{m}^{3}\cdot MPa}\right]=\frac{{C}_{\infty }\mathrm{ slope }\left[\frac{wt\cdot ppm}{MPa}\right]\times {10}^{6}\times d\left[\frac{g}{{m}^{3}}\right]}{{m}_{g}\left[\frac{g}{mol}\right]},$$

where *m*_g_ is the molar mass of gas used and *d* is the density of polymers. The permeability of four gases in the NBR, EPDM, LDPE and HDPE polymers is obtained from the solubility and the average diffusivity by using the relation P = D_ave_S. The permeation parameters for the four gases in NBR, EPDM, LDPE and HDPE are summarized and compared with those obtained by different methods in Table 2.

**Table 2:** Summary of permeability parameters for the four gases in NBR, EPDM, LDPE and HDPE.

Specimen	Solubility (mol/m^3 ·^MPa)				Diffusivity (× 10^–11^ m^2^/s)				Permeability (mol/m s MPa, × 10^–10^)			
	H_2_	He	N_2_	Ar	H_2_	He	N_2_	Ar	H_2_	He	N_2_	Ar
NBR	34.2 (35.3)^a^	8.96	11.0	22.5	5.60 (6.50)^a^	21.5	1.18	2.01	19.2 (22.8)^a^	19.3	1.29	4.53
EPDM	25.6 (26.2)^21^	7.79	17.0	38.6	19.7 (24.1)^21^	83.1	7.24	10.5	50.3 (63.1)^21^	64.8	12.3	40.4
LDPE	5.38	1.55	7.25	15.7	4.17	91.4	2.53	4.78	2.24	14.2	1.83	7.49
HDPE	3.68	1.08	3.67	7.43	4.76	78.3	2.69	5.30	1.75	8.5	0.99	3.94

^a^Is determined by differential pressure method.

The value in parentheses is determined by the differential pressure method and thermal desorption analysis-GC^21^ in the same specimen. The H_2_ results obtained by different methods are consistent with those obtained in the present work. The procedure/method for determining S, D and permeability (P) in GC is similar as volumetric method, although the principle/apparatus for measuring S and D is differ each other. However, the differential pressure method has different sequence for obtaining the permeation parameters as volumetric method. The S is finally determined as $\mathrm{S}=\frac{P}{D}$ from D by lag time measurement and P by slope measurement of pressure change with regards to elapsed time.

The uncertainty factor and relative expanded uncertainty of measurement for gas diffusion properties are represented in Table 3. The dominant uncertainty factors in the solubility and diffusivity measurement are mainly due to the repeated measurements, standard deviation between data and Eqs. (5)–(7), volume change of specimen in the pressurization/decompression process. The type A uncertainty resulting from repeated measurements is obtained by three times measurements.

**Table 3:** Summary of uncertainty factor and relative expanded uncertainty for solubility and diffusivity of gas.

Uncertainty factor	Value (%)
Repeated measurements	4.2
Standard deviation between data and Eqs. (5)–(7)	1.7
Volume change of specimen	1.7
Calibration certificate of electronic balances	0.001
Accuracy of graduated cylinder	0.3
Resolution of graduated cylinder	0.2
Accuracy of manometer	0.6
Combined standard uncertainty, *u*_c_	4.9
Coverage factor, *k*	2.1
Expanded uncertainty, *U* = *ku*_c_	10.3

Meanwhile, the type B uncertainty contributions, except for the graduated cylinder resolution, are obtained by dividing a factor of $\sqrt{3}$ with an assumption of a rectangular distribution. The standard deviation between the data of H_2_ content versus elapsed time and the least squared fit obtained using Eqs. (5) to (7) is less than 3%. Considering that the maximum deviation is 3%, the type B uncertainty can be obtained as 1.7%. The volume change of specimen in the pressurization/decompression process is measured as less than 3%, the type B uncertainty can be obtained as 1.7%. For measurement of sample mass, the uncertainty of calibration certificate of electronic balances amounts to 0.001%. The accuracy of graduated cylinder is 0.5%, thus the type B uncertainty obtained is 0.3%. When the graduated cylinder of 10 ml is used, the minimum readable scale is 0.1 ml, which corresponds to uncertainty of 1%. The resolution is half of this minimum value. Thus, the type B uncertainty by the resolution is obtained as 0.2% by dividing the factor of $\sqrt{6}$ using a triangular probability distribution. The accuracy of the analog manometer is 1%, which corresponds to GRADE A. Therefore, the type B uncertainty can also be obtained as 0.6%. The variations of both temperature and pressure during the measurement in the laboratory are removed by considering their change.

The combined standard uncertainty is expressed as a root sum of squares of the uncertainty source. The relative expanded uncertainty is obtained by assuming a normal distribution and multiplying the combined standard uncertainty by a coverage factor of 2.1 at a 95% confidence level. The relative expanded uncertainty for solubility and diffusivity is not more than 10.3%.

**Figure 10:**
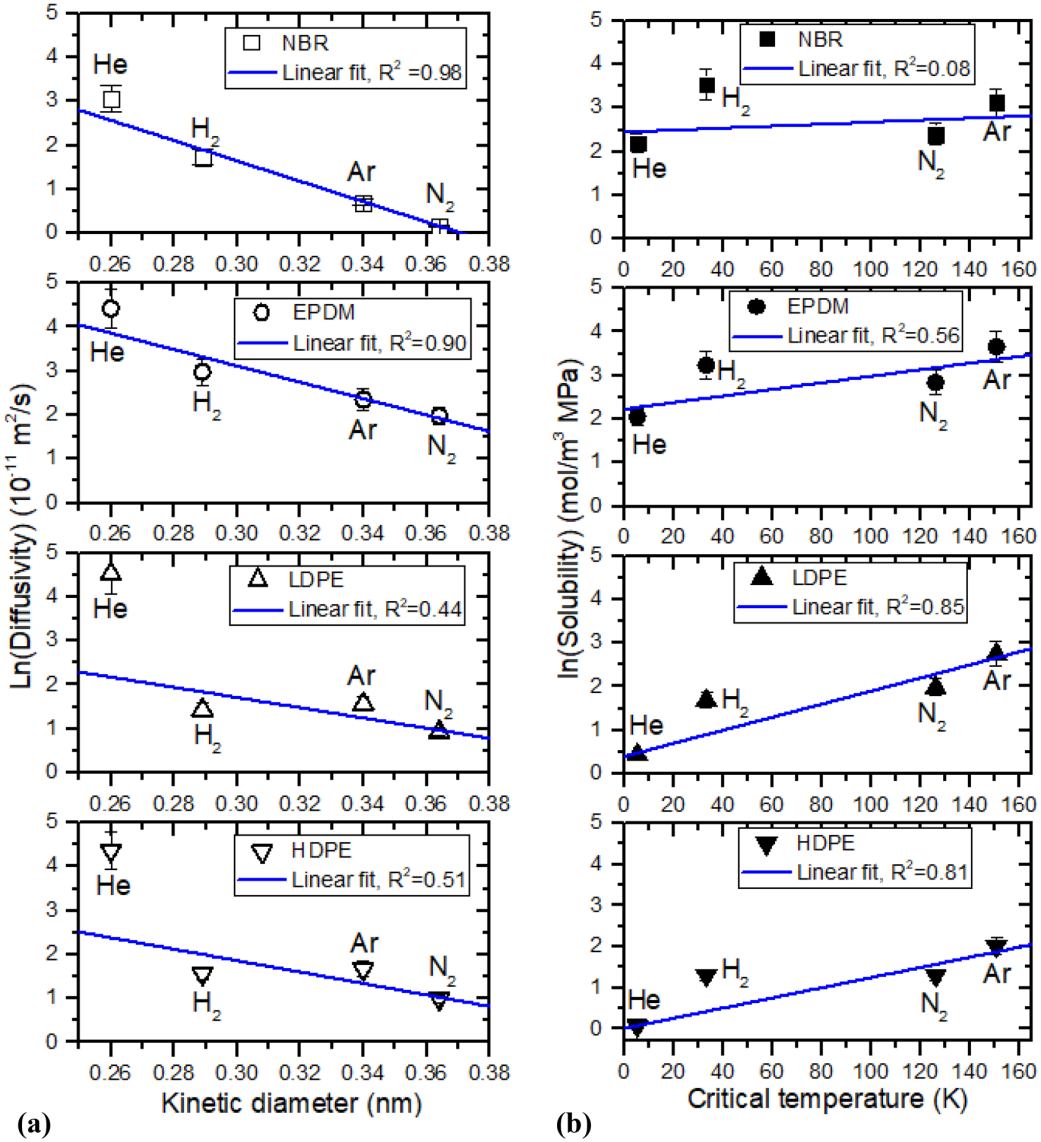
Linear correlation between (**a**) logarithmic diffusivity and kinetic diameter, (**b**) logarithmic solubility and critical temperature of gas molecules in NBR, EPDM, LDPE and HDPE polymers.

Figure 10 shows the diffusivity and solubility determined by three-channel volumetric measurement for four polymers. In summary, the diffusion parameters *C*_∞_ and D are determined using a diffusion analysis program by the application of Eqs. (5) to (7). The S is determined from linear slope for uptake (*C*_∞_) versus pressure by Eq. (8). The P of four gases in the NBR, EPDM, LDPE and HDPE polymers is finally obtained from the S and the average D.

The difference in permeation parameters depending on gases was obviously found in NBR, EPDM, LDPE and HDPE. The magnitude of the diffusivity decreases in the order *D*_He_ > *D*_H2_ > *D*_Ar_ > *D*_N2_ in all NBR, EPDM, LDPE and HDPE specimens. For diffusivity analysis, we focus on the effective molecule size of gas rather than the molar mass of gas. The size of the permeant molecule affects diffusivity. As the effective size of the molecule increases, the diffusivity decreases. As expected, we found a linear correlation between the logarithmic diffusivity and kinetic diameter of molecules in gas for NBR, EPDM, LDPE and HDPE, as indicated by the blue line on the left side of Fig. 10. The kinetic diameters of He, H_2_, Ar and N_2_ molecules are 0.26 nm, 0.29 nm, 0.34 nm and 0.364 nm, respectively. Kinetic diameter is the size of the sphere of influence that can lead to a scattering event and is related to the mean free path of molecules in a gas^22,23^. As shown in Fig. 10, He gas in LDPE and HDPE deviates from linearity. The diffusivity tends to decrease with increasing kinetic diameter of the molecule. The variation in the logarithmic diffusivity of He, H_2_, Ar and N_2_ varied linearly versus the kinetic diameter of gases for all polymers and was in good agreement with previously reported results^24–26^.

Meanwhile, Fig. 10b shows the solubility of the gas molecule for four polymers. The magnitude of the solubility in NBR decreases in the order S_H2_ > S_Ar_ > S_N2_ > S_He_. The magnitude of the solubility in EPDM decreases in the order S_Ar_ > S_H2_ > S_N2_ > S_He_. The magnitude of the solubility in LDPE decreases in the order S_Ar_ > S_N2_ > S_H2_ > S_He_. The magnitude of the solubility in HDPE decreases in the order S_Ar_ > S_H2_ > S_N2_ > S_He_.

It was reported that gas sorption content in polymeric membranes depends on the condensability-related to the critical temperature of gases, interaction between the polymer and gas molecules, crystallinity of the polymer, temperature and pressure^26,27^. Although there are factors related with gas permeation, the gas solubility is related with the critical temperature (T_c_) as^28,29^.

9 $${\text{ln}}\,{\text{S }} = {\text{ ln S}}_{0} + {\text{ KT}}_{{\text{c}}} ,$$

where S_0_ and K are constants. The critical temperature is a measure of condensation for gaseous molecules. As expected, it is observed that the logarithmic solubility increases nearly linearly with the increasing the critical temperature in Fig. 10b, except for H_2_ gas deviation from linearity. The similar relationship was reported for polyvinylpyridine film^29^.

A linear correlation with relatively good R^2^ for both LDPE/HDPE rather than both NBR/EPDM was found. In particular, the solubility of He gas in all polymers investigated in this work is smallest and is in contrast to the diffusivity magnitude. The fast diffusion and small solubility observed in He seem to be different behaviors, which is unlikely for other gases. The small solubility of He is attributed to its small condensability, known as 5.19 K^30^, which is the critical temperature of the He gas molecule. This value is smaller than the values of H_2_ and N_2_. However, the origin of the solubility difference will be clarified in future research.

## Conclusions

We determined the permeation properties of various gases, H_2_, He, N_2_ and Ar for four polymers, with precise volumetric analysis measurements using a graduated cylinder and updated diffusion analysis program, which could be commonly applicable for various shaped-specimen. The technique simultaneously and parallel evaluates three sets of diffusion characteristics of gas by quantitatively analyzing the amount of gas released after high-pressure gas charging and subsequent decompression. Compensating the variation in temperature and pressure, the fluctuation obtained by varying the temperature and pressure of the laboratory environment was removed. The investigated results obtained for the polymers demonstrate that the permeation properties of H_2_ determined by the developed method are in good agreement with those determined by the differential pressure method and gas chromatography.

The experimental investigation indicates that the gas content emitted from NBR, EPDM, LDPE and HDPE satisfied Henry’s law up to a pressure of 9 MPa, which confirmed that the content was primarily proportional to the pressure. The solubilities and diffusivities in all specimens employed are identical, regardless of the sample shape and dimension. This is a general trend as expected.

Meanwhile, the diffusivity decreases in the order *D*_He_ > *D*_H2_ > *D*_Ar_ > *D*_N2_ in four specimens. The different diffusivities depending on gas species are attributed to their different kinetic diameters, related to the mean free path of gas molecules. The linear relationship between logarithmic solubility and critical temperature of gas used are new observation. Especially, the small He gas solubility is attributed to the small critical temperature of the gas.

In conclusion, a technique for determining permeation with volumetric measurement by graduated cylinders could be effectively applied to automatically evaluate the transport properties of gases in polymers and other materials for cases requiring real-time and time-consuming measurements with small diffusion coefficients. This simple technique could be commonly applied to permeation evaluation for all kinds of gas, irrespective of sample size, shape and gas species.
